# Influence of basic knowledge about female health, physiology, and contraception on unintended pregnancies: an international multicenter survey among women in Austria, Germany and Switzerland

**DOI:** 10.1007/s00404-024-07441-1

**Published:** 2024-03-04

**Authors:** Christina Allerstorfer, Elisabeth Reiter, Omar Shebl, Peter Oppelt, Andrea Müller Reid, Wolfgang Schimetta, Helge Binder, Matthias W. Beckmann

**Affiliations:** 1 Department of Gynecology, Obstetrics and Gynecological Endocrinology, Kepler University Hospital, Johannes Kepler University Linz, Altenberger Strasse 69, 4040 Linz, Austria; 2 Faculty of Medicine, Johannes Kepler University Linz, Linz, Austria; 3 Frauenpunkt, Altdorf, Switzerland; 4 Department of Applied Systems Research and Statistics, Johannes Kepler University Linz, Linz, Austria; 5 Department of Obstetrics and Gynecology, Friedrich Alexander University of Erlangen–Nuremberg (FAU), Erlangen, Germany

## Introduction

Many publications confirm that a large number of pregnancies worldwide are unplanned. According to the European Parliamentary Forum on Population and Development, 43% of pregnancies in Europe are unintended [1]. Worldwide, there are 121 million unintended pregnancies annually [2]. Although the numbers of unintended pregnancies have declined in recent years [3], unwanted pregnancies still lead to 43.8 million abortions per year worldwide [4]. Unreported cases may be much higher. According to Sedgh et al., 50% of all unplanned pregnancies in 2012 led to induced abortions, 13% ended in miscarriage, and only 37% resulted in live births [3].

Unintended pregnancies not only have physical implications, they are also subsequently associated with poorer mental health conditions among the offspring [5]. According to a British study, there are three main reasons for unplanned pregnancies: incorrect application of contraceptives, a lack of knowledge about emergency contraception, and poor information provided by medical personnel [6].

With regard to sociodemographic status in relation to unintended pregnancies, some studies have reported that younger women aged between 18 and 29 years are more prone to unwanted pregnancies [7]. A second peak in unwanted pregnancies was observed in women over the age of 40. Surprisingly, more than 50% of pregnancies in women aged over 40 appear to be unintended [8]. There was also a higher risk of unintended pregnancies among ethnic minorities, unmarried women, and women with a low income [9].

The aim of the present study was to evaluate women’s level of knowledge about contraception and sexual health in the three German-speaking countries—Austria, Germany, and Switzerland—and to identify whether there are any negative or positive correlations between responses to the survey questions and the frequency of unintended pregnancies, as well as the individual participants’ sociodemographic status in the three different countries.

## Materials and methods

Approval for the study was obtained from the ethics committee for Kepler University Hospital in Linz (Ethics Committee for the State of Upper Austria) on August 23, 2012 (ref. no. L-4-12).

Hospitals in three different countries took part in this international multicenter survey: Kepler University Hospital Linz (Austria), Erlangen University Hospital (Germany), and Uri Cantonal Hospital (Altdorf, Switzerland). Only gynecological and obstetric outpatients aged between 15 and 50 years were included in the study. Patients who had language barriers preventing them from completing a questionnaire were excluded. A validated questionnaire was used to evaluate the participant’s knowledge about contraception and sexual health and to obtain information about her sociodemographic status. The questionnaires were completed during the waiting time before appointments with the physician. The goal was to obtain at least 200 completed questionnaires per center.

A two-tiered questionnaire was used. The first part identified the participant’s sociodemographic characteristics. Information about the following parameters was requested: nationality; current pregnancy status; age (years); immigration background; educational level; income above €10,000 (CHF 18,000; including part-time employment or unemployment); pregnancy history: number of all pregnancies and of unintended pregnancies, number of previous induced abortions, number of previous miscarriages/stillbirths, number of children, number of cesarean sections and vaginal deliveries; and marital status (married, legally separated, widowhood, single, in partnership).

The second part of the questionnaire was a shortened version of a standardized questionnaire developed by David et al. [10], with specific questions regarding participants’ knowledge of female anatomy and physiological processes, and about their sources of information. The questionnaire consisted of 10 questions about contraception, sexual health, anatomy, physiology, and pregnancy prevention. One point was scored for each correctly answered question, so that a maximum of 10 points could be obtained (see “Appendix”).

### Statistical analysis

Two-sided 95% confidence intervals (95% CI) were calculated for responses to the items listed in the questionnaire and the score values calculated. Forward stepwise multiple regression analysis was carried out, with the score value as a dependent variable. Spearman’s rank correlation coefficients or nonparametric, point biserial correlation coefficients were calculated for the score and sociodemographic characteristics as independent variables. Nonparametric variance analysis (Kruskal–Wallis analysis) was used to compare metric and ordinal variables obtained in the three centers. Categorical qualitative variables were compared using the chi-square test.

Multiple testing was not adjusted for type I errors. The resulting *P* values are therefore only descriptive. The open-source R statistical software package, version 3.0.1 (Institute for Statistics and Mathematics, University of Vienna, Austria), was used for statistical analysis.

## Results

A total of 605 completed questionnaires were obtained: 203 from Austria, 202 from Germany, and 200 from Switzerland. The women’s mean age was 32.96 ± 9.12 years; 54% were married, 21.5% were living in a partnership, and 19.3% were single; 44.8% had completed upper secondary school education; and 63.5% stated that their net income was over € 10,000 / CHF 18,000 (Table 1).

**Table 1:** Selected sociodemographic characteristics relative to each country

Center	Age (year)	Married (%)	In partnership (%)	Single (%)	Upper secondary school education (%)	Annual net income > € 10,000/CHF 18,000 (%)
Austria	31.92 (± 9.08)	48.8	24.1	19.2	51.7	55.7
Germany	33.18 (± 8.17)	53.3	26.7	17.3	52.9	72.7
Switzerland	33.79 (± 10)	60.0	13.5	21.5	44.8	63.5

Electronic or paper media were the main source of the information that the patients had in all three test centers. In Germany and Switzerland, family and friends were the second most important source of information, whereas in Austria schools were the second most important source of knowledge. Physicians played a minor role here in all three countries (Table 2).

**Table 2:** Most relevant sources of information about female health, physiology, and contraception among the participants

	Austria	Germany	Switzerland
1	Media (80.3%)	Media (82.7%)	Media (81.8%)
2	Education (72.4%)	Family and friends (71.8%)	Family and friends (75.0%)
3	Family and friends (71.4%)	Education (68.8%)	Education (73.5%)
4	Others (4.9%)	Physicians (5.0%)	Others (10.0%)
5	Physicians (3.4%)	Others (4.5%)	Physicians (2.0%)

**Fig. 1:**
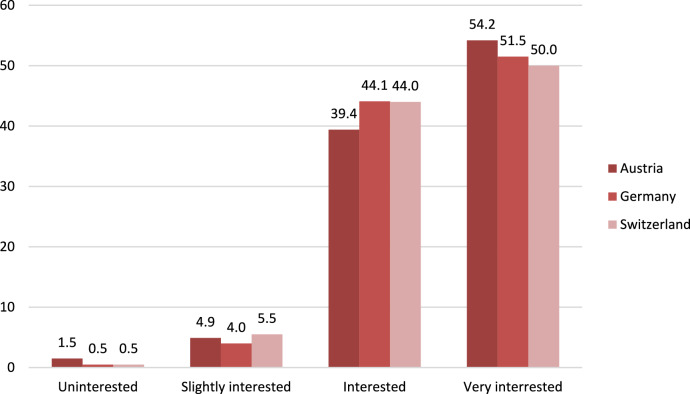
Level of interest in female physiology among the participants

More than 90% of the women were interested or very interested in understanding the physiological processes of the female reproductive system, with only marginal differences between the countries. Only 0.8% of the women interviewed expressed no interest in the topic at all (Fig. 1).

When asked to assess their level of knowledge about female anatomy and physiology, only 12.7% of the participants stated that they had a very good level. The great majority of them regarded their level of knowledge as good (62.0%), while only 19.3% thought that it was satisfactory, 5.6% said it was adequate, and only 0.3% regarded their knowledge level as insufficient.

The participants had a mean score of 7.57 ± 1.83 points in the knowledge test. Austrian women had slightly lower scores (7.43 ± 1.85), and German women had slightly higher scores (7.68 ± 1.93). The mean score for the participants in central Switzerland was between these two levels (7.59 ± 1.71).

**Fig. 2:**
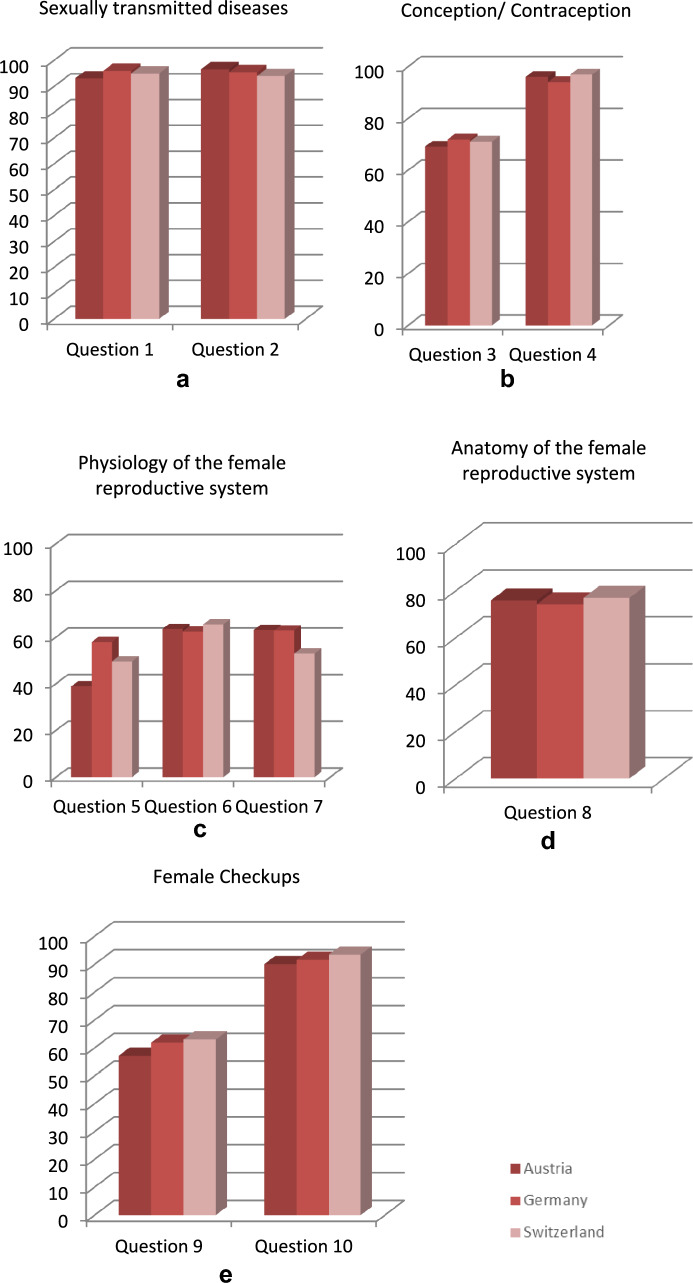
Correctly answered questions per category, in percent (questions are listed in the Appendix). **a** Questions 1 & 2: sexually transmitted diseases. **b** Questions 3 and 4: conception and contraception. **c** Questions 5 and 6: physiology of the female reproductive system. **d** Question 8: anatomy of the female reproductive system. **e** Questions 9 and 10: check-ups for gynecological cancer surveillance

Closer examination of the test data showed that the participants had varying levels of knowledge about certain topics. While more than 90% of the women were able to answer the questions about sexually transmitted diseases (STDs) correctly (questions 1 and 2), the question about the fertile phase during the female menstrual cycle (question 3) was answered correctly by fewer women (70.6%). The questionnaire revealed gaps in the participants’ knowledge regarding the physiological processes of menstruation and ovulation (questions 5–7). Fewer than 50% were familiar with the hormonal changes causing menstrual bleeding. There was a good level of knowledge about contraceptives and the anatomy of the female genital organs (questions 4 and 8). Ninety percent were able to answer these two questions correctly. Two-thirds of the participants were less well informed about check-ups for gynecological cancer surveillance (questions 9 and 10) (Fig. 2a–f).

In the group of 605 women interviewed, a significant correlation was observed between unintended pregnancies and the participants’ level of knowledge about contraception. In women with previous unintended pregnancies, there was also a significantly lower level of knowledge in comparison with women who had not had any unintended pregnancies (*P* = 0.024).

With regard to sociodemographic status, the following factors were found to influence the level of knowledge: nationality (*P* < 0.001), age (*P* < 0.001), income (*P* < 0.001), educational level (*P* < 0.001), marital status (*P* < 0.001), previous planned pregnancies (*P* = 0.004), previous unplanned pregnancies (*P* = 0.024), previous induced abortions (*P* = 0.027), and previous miscarriages or stillbirths (*P* = 0.004). All of these parameters showed significant or very significant *P* values (Table 3).

**Table 3:** Correlation between sociodemographic factors and level of knowledge (multiple regression analysis)

	Coefficient	T score	*P* value
Nationality	1.839	7.897	< 0.001**
Educational level	0.811	6.064	< 0.001**
Marital status	0.608	4.059	< 0.001**
Age (years)	0.028	3.253	0.001**
Annual net income more than EUR 10.000/ CHF 18.000	0.288	2.000	0.046*
Abortions/ stillbirths (n)	0.350	2.745	0.006**
Previous pregnancies (yes/no)	– 0.368	– 2.197	0.028*

*Significant, **very significant

Multiple regression analysis showed that a married 20-year old woman with an annual net income above € 10,000 who had completed upper secondary school education and did not have any previous pregnancies reached a cumulative score of 8.318. By contrast, a 20-year old woman with an immigrant background and an annual net income of less than € 10,000, with no access to upper secondary school education, who had been unintentionally pregnant more than once and also had at least one abortion, reached a low cumulative score of 4.754.

## Discussion

These findings are comparable with the results of recent studies reporting gaps in women’s knowledge about contraceptive options. Although a few studies have evaluated the level of knowledge in this area among Austrian adolescents [11, 12], adult Austrian women have only been interviewed in one study so far. The results of the study showed that almost 50% of women did not know how oral contraceptives actually work. Most of them were convinced that oral contraceptives have serious and harmful side effects [13]. A study conducted by David et al. among German and Turkish women regarding contraception and physiological processes showed knowledge gaps similar to those in the present study [10]. Only 44.8% of the German women interviewed and 22.7% of the Turkish women were able to positively identify the fertile phase during a woman’s menstrual cycle. When asked about which hormonal changes induce menstrual bleeding, the results were even poorer: 39.5% of the German women and 12.8% of the Turkish women knew the correct answer, roughly the same level as in the present study [10].

A correlation was observed between sociodemographic status and the level of knowledge about contraception. The women in the German study center achieved the best scores (7.68), while women in the other participating centers had lower ones. These differences may in part be explained by certain sociodemographic differences. German women were more likely to have German citizenship and a higher level of school education. They also had higher incomes and fewer previous pregnancies. By contrast, women in the Austrian study center more often had an immigrant background, a lower level of school education, were earning less and had been pregnant more often. All of these factors, as well as others, have an influence on the level of knowledge.

Only one study, conducted in Switzerland, has reported results comparable with the above findings. Women in the study were able to name more contraceptive methods if they were well educated and had a good socioeconomic status [14].

In all of the countries included in this study, the media were women’s main source of information about contraceptive options (Table 2). This underlines the importance of the Internet, magazines, and other types of media in providing adequate sexual education. It contrasts with the findings of an earlier study among Austrian adolescents, in which the main source of information was found to be medical professionals and the women’s partners [13]. The study is only poorly comparable with the present survey, however, as it was published more than 30 years ago. It can be assumed that digital media are much more important nowadays, as they are easily and anonymously accessible.

In another study, Loeber et al. argue that women with multiple unintended pregnancies should be provided with access to professional counseling about better contraceptive usage [15].

The present study may be subject to a certain degree of bias, as the questionnaire was handed out in a waiting room with other patients present. It is not known whether the participants had any help from an accompanying or neighboring person. Nevertheless, it is the first multicenter and three-country study investigating the level of women’s information about the use of contraceptives and their knowledge of female physiology and sexually transmitted diseases, correlated with sociodemographic status and data on unintended pregnancies.

## Conclusion

These results show that most of the women who took part were well informed about sexually transmitted diseases, but that there were gaps in their knowledge concerning the anatomy and physiology of the female reproductive system. This is an important finding, as a statistically significant correlation was found between the level of knowledge and unintended pregnancies. Statistically significant correlations with some sociodemographic factors were also noted. The present study underlines the importance of providing women with adequate counseling in order to avoid unintended pregnancies and induced abortions.

## Data Availability

Data available upon request.

## Appendix: scored items in the questionnaire

**Table:**

Question 1:	How can sexually transmitted diseases be prevented?
	Correct answer (= 1 point): By using condoms.
	Wrong answers (= 0 points): Not having intercourse too frequently; taking the oral contraceptive pill; daily washing of the vagina with a mild lotion is best; I don’t know.
Question 2:	What is the most common route for transmission of a sexually transmitted disease?
	Correct answer (= 1 point): Via semen and vaginal fluid.
	Wrong answers (= 0 points): Through the use of public toilets; by drinking from a shared glass; via skin contact when shaking hands; I don’t know.
Question 3:	When is conception very unlikely in women with a regular menstrual cycle?
	Correct answer (= 1 point): In the first and last weeks of the 4-week menstrual cycle.
	Wrong answers (= 0 points): 6 days before until 6 days after ovulation (middle of the menstrual cycle); if she has sexual intercourse while standing; during ovulation (the hormones “kill” all sperm); I don’t know.
Question 4:	Which options for contraception do you know about?
	Correct answers (3 correct answers = 1 point): condom; contraceptive pill; intrauterine device; contraceptive patch; contraceptive injection; sterilization; …
Question 5:	Menstrual bleeding is …
	Correct answer (= 1 point): Withdrawal bleeding, because the crucial hormone levels are dropping.
	Wrong answers (= 0 points): Bleeding that occurs because the level of hormones in the blood is raised; bleeding caused by injury during sexual intercourse; bleeding caused by implantation of the ovum; I don’t know.
Question 6:	What happens during ovulation?
	Correct answer (= 1 point): The matured ovum passes from the ovary into the fallopian tube.
	Wrong answers (= 0 points): The ovum is formed in the fallopian tube and “jumps“ into the uterus; the ovum in the ovary “bursts“ to deliver the finished embryo to the uterus; the ovum is expelled from the ovary and excreted through the urine; I don’t know.
Question 7:	How does body temperature change after ovulation?
	Correct answer (= 1 point): The temperature rises.
	Wrong answers (= 0 points): The temperature drops; the temperature does not change; no answer, as there is no connection between ovulation and body temperature; I don’t know.
Question 8:	Indication of the internal female genital organs on the basis of an illustration.
	Correct answer (= 1 point), wrong answers (= 0 points).
Question 9:	What is the examination method called that is used to check whether the cells of the cervix and vagina are normal, or if they are showing any abnormalities?
	Correct answers (= 1 point): PAP smear, cancer smear, cervical smear
Question 10:	What is a mammography?
	Correct answer (= 1 point): X-ray examination of the breast.
	Wrong answers (= 0 points): Ultrasound of the abdomen; ultrasound of the breast for early tumor detection; biopsy of breast tissue; I don’t know.
